# From the Editor's Desk

**Published:** 2014

**Authors:** Patrick Commerford

**Affiliations:** Professor Emeritus, Senior Scholar, Cardiac Clinic, Department of Medicine, University of Cape Town

I am delighted to have been appointed Editor-in-Chief of the *Cardiovascular Journal of Africa* and believe it appropriate in this first issue in which I am involved to pay tribute to my predecessors, to acknowledge my previous error and express my hope that I can continue the traditions of the Journal.

When the late Professor Andries Brink approached me as a newly appointed head of Cardiology at the University of Cape Town and Groote Schuur Hospital in the late 1980s with his innovative proposal for a uniquely South African cardiology journal, I was not enthusiastic. I responded with youthful arrogance and ignorance that ‘there were more than enough journals and we did not need another one’. How wrong that was!

Professor Andries Brink forged ahead, despite widespread scepticism, founded the *Cardiovascular Journal of South Africa* and under his guidance and rigorous scholarship it became established and recognised as a repository for publications of quality, initially mainly from South Africa, but with increasing numbers from other countries. When Professor Andries Brink passed away, his son Professor Paul Brink single-handedly continued to edit and direct the Journal. I consider that the highpoint of its 25-year growth trajectory was the acceptance of the Journal as the official journal of the Pan-African Society of Cardiology (PASCAR) and its change of name to the current one of the *Cardiovascular Journal of Africa*.

In 2014 it is difficult for those who did not personally experience the events of the decades prior to 1994 to understand or appreciate exactly how important the changes and developments post 1994 have been. In the 1980s, academic, clinical and scientific contact between South African cardiologists and our colleagues in most of the rest of Africa was negligible. South Africans faced academic boycotts from countries both within and without Africa. Travel to African countries for South Africans was extraordinarily difficult and it was equally difficult, if not more so, for colleagues from other African countries to visit South Africa. How wonderful to have experienced how things have changed for the better!

Today there is vigorous interchange and interaction, with collegial participation at continental meetings, exchanges of trainees, and a generous willingness to share expertise throughout the continent. Very importantly, multicentre co-operative research into the diseases of Africa has been instituted, and hopefully in the near future, new insights facilitating the management of patients with diseases that are common in Africa will become available. Other multicentre epidemiological investigations and registries on the impact of conditions not previously investigated in depth in Africa, such as coronary artery disease and atrial fibrillation, have been remarkably successful. The Journal has been involved directly or indirectly in much of this.

The two Professors Brink, Andries and Paul, deserve recognition for their major contributions to clinical medicine, cardiology and cardiovascular science, but very importantly also, for their role in helping to develop and grow a continental publishing initiative that is enhancing patient care, medical training, and cardiovascular research in Africa.

In this editorial I have tried to highlight some items of particular interest to me in the current issue. This is not comprehensive and inevitably driven by personal preference.

Many states in Africa lack reliable information on health and illness issues, which would allow planners to allocate scarce resources to areas of need, and healthcare practitioners to examine and improve outputs. There are new and very helpful efforts to correct this problem. In this issue Bonny and colleagues (page 176) provide an overview of sudden cardiac death in Africa and an ambitious proposal to collect reliable information. Chin (page 151) provides an editorial comment summarising the problem and potential solutions. The outcome of the project will be eagerly awaited. In a similar vein, Brun and colleagues (page 159) examine a large number of patients at risk of venous thromboembolism and demonstrate under-use of recommended prophylactic anticoagulation. These two initiatives are both multicentre studies involving sites from many different African countries and it is heartening to see such co-operation, which can only bode well for the future.

In a prospective cohort study, Borkum and co-workers (page 153) examined ambulatory blood pressure and renal function in asymptomatic HIV-positive patients. The prevalence of chronic kidney disease was lower than anticipated and HIV infection was associated with an ambulatory non-dipping status, which suggests an underlying dysregulation of the cardiovascular system. In the short term, anti-retroviral therapy did not seem to improve loss of circadian rhythm. A non-dipping pattern is an established entity with clinical implications, and is associated with higher cardiovascular morbidity and mortality rates. The high prevalence of non-dippers in the HIV-infected group in this study supports data from other countries. How this non-dipping pattern will impact on long-term mortality and morbidity in HIV-positive patients remains to be established.

With the increasing and widespread use of new imaging modalities, we are learning more about cardiac function and involvement in systemic diseases. An article from Turkey (page 168) describes echocardiographic abnormalities in patients with rheumatoid arthritis and the influence of treatment on those abnormalities. Interestingly for me, the old-fashioned but evergreen ECG also reflected some of the changes. Cardiac imaging is further highlighted in a review of cardiac magnetic resonance imaging by Scholtz and others (page 185). This reports the clinical utility of this exciting modality.

The letter from Schamroth (page 192) regarding the adverse effects of the Noakes diet on lipids is of considerable interest and concern, given several such anecdotal verbal reports from colleagues around South Africa.

